# The Extinction of Dengue through Natural Vulnerability of Its Vectors

**DOI:** 10.1371/journal.pntd.0000922

**Published:** 2010-12-21

**Authors:** Craig R. Williams, Christie A. Bader, Michael R. Kearney, Scott A. Ritchie, Richard C. Russell

**Affiliations:** 1 Sansom Institute for Health Research, University of South Australia, Adelaide, South Australia, Australia; 2 Department of Zoology, The University of Melbourne, Melbourne, Victoria, Australia; 3 School of Public Health, Tropical Medicine and Rehabilitation Sciences, James Cook University, Cairns, Queensland, Australia; 4 Department of Medical Entomology, Sydney Medical School, University of Sydney and Westmead Hospital, Sydney, New South Wales, Australia; USAMRIID, United States of America

## Abstract

**Background:**

Dengue is the world's most important mosquito-borne viral illness. Successful future management of this disease requires an understanding of the population dynamics of the vector, especially in the context of changing climates. Our capacity to predict future dynamics is reflected in our ability to explain the significant historical changes in the distribution and abundance of the disease and its vector.

**Methodology/Principal Findings:**

Here we combine daily weather records with simulation modelling techniques to explain vector (*Aedes aegypti* (L.)) persistence within its current and historic ranges in Australia. We show that, in regions where dengue presently occurs in Australia (the Wet Tropics region of Far North Queensland), conditions are persistently suitable for year-round adult *Ae. aegypti* activity and oviposition. In the historic range, however, the vector is vulnerable to periodic extinction due to the combined influence of adult activity constraints and stochastic loss of suitable oviposition sites.

**Conclusions/Significance:**

These results, together with changes in water-storage behaviour by humans, can explain the observed historical range contraction of the disease vector. For these reasons, future eradication of dengue in wet tropical regions will be extremely difficult through classical mosquito control methods alone. However, control of *Ae. aegypti* in sub-tropical and temperate regions will be greatly facilitated by government policy regulating domestic water-storage. Exploitation of the natural vulnerabilities of dengue vectors (e.g., habitat specificity, climatic limitations) should be integrated with the emerging novel transgenic and symbiotic bacterial control techniques to develop future control and elimination strategies.

## Author Summary

Dengue transmission has not always been confined to tropical areas. In some cases, this has been due to a reduced geographic range of the mosquitoes that are able to carry dengue viruses. In Australia, *Aedes aegypti* mosquitoes once occurred throughout temperate, drier parts of the country but are now restricted to the wet tropics. We used a computer modelling approach to determine whether these mosquitoes could inhabit their former range. This was done by simulating dengue mosquito populations in virtual environments that experienced 10 years of actual daily weather conditions (1998–2007) obtained for 13 locations inside and outside the current tropical range. We discovered that in areas outside the Australian wet tropics, *Ae. aegypti* often becomes extinct, particularly when conditions are too cool for year-round egg-laying activity, and/or too dry for eggs to hatch. Thus, despite being a global pest and disease vector, *Ae. aegypti* mosquitoes are naturally vulnerable to extinction in certain conditions. Such vulnerability should be exploited in vector control programs.

## Introduction

Dengue fever is a public health problem of global importance, producing a spectrum of disease spanning febrile arthralgia to hemorrhagic death. Dengue viruses are transmitted between human hosts almost exclusively by *Aedes (Stegomyia) aegypti* and *Aedes (Stg.) albopictus* mosquitoes, both of which are well adapted to using artificial containers for larval habitat. Many urban areas in the tropical world are subject to dengue transmission [1], the geographic range of which is limited by the distribution of the vectors. However, these ranges are not static, with numerous expansions and retractions recorded through time. Despite great progress in the development of novel control techniques for *Ae. aegypti* [2], [3], our understanding of how dengue and its vectors become extinct is poor.

The principal vector, *Ae. aegypti*, is thought to have originated in Africa and extended its range globally with the expansion of commercial shipping in the 17^th^ and 18^th^ centuries [4], [5]. While this range was significantly reduced by numerous eradication programs in the Americas from the 1930s to the 1970s [6], [7], *Ae. aegypti* soon regained much of its former range after these programs ceased [7].

An ultimate cause of such range plasticity is human activity. The production of suitable larval habitats (i.e. artificial containers) and human-facilitated transport has encouraged the dispersal and establishment of these mosquitoes. Increased urbanisation without properly planned waste management and water handling systems has also created ideal conditions for mosquito breeding [7]. Human activity is thus a key determinant of dengue vector populations.

**Figure 1:**
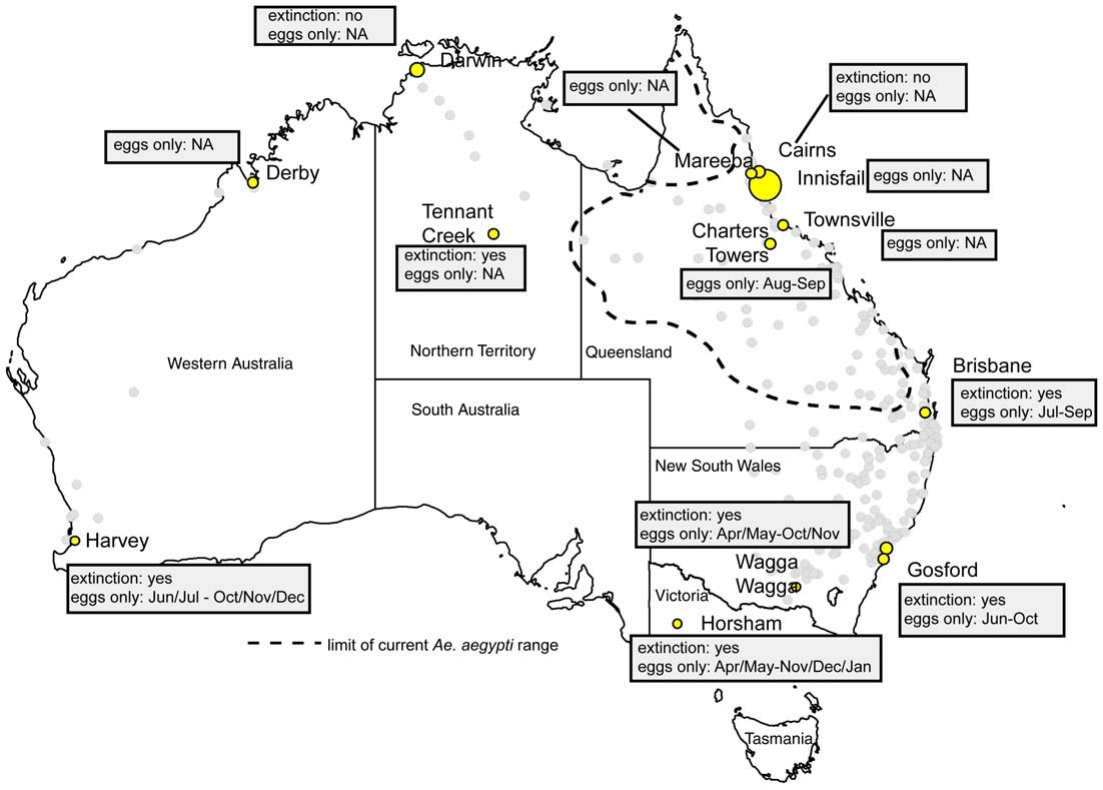
*Aedes aegypti* persistence and performance at localities throughout the historic (faint grey circles) and current range (indicated by dashed line) in Australia. Size of yellow circles indicates mean egg abundance per ha. for simulated localities. ‘Extinction yes/no’ refers to whether or not extinction was detected during simulations (not tested for all localities). ‘Eggs only’ refers to time of year when *Ae. aegypti* reduced to eggs as only life-stage present. NA  =  not applicable.

In Australia, dengue transmission is currently restricted to tropical north Queensland (Qld) (Fig. 1). The vector there, *Ae. aegypti*, is most abundant and active year-round in the tropics, yet its distribution extends into sub-tropical coastal central Qld, and some arid inland areas [8]. Dengue has been recorded in Australia from as early as 1873 [9], and although outbreaks have been most common in the tropics, sporadic activity has also occurred in the subtropics and temperate regions [10]. This was due in part to the distribution of *Ae. aegypti* formerly extending well into temperate regions (up to 33°S in Western Australia (WA)). However, a range retraction occurred in the last half of the 20^th^ century, with the last collections from New South Wales (NSW) in 1948, and WA in 1970. The last records from the Northern Territory (NT) were from 1956 [11], with established (incursant) *Ae. aegypti* populations not discovered again until 2004 and 2006 [12]. It also disappeared from southern Qld in the 1950s. The current range has been relatively stable for the last three decades at least.

The cause of *Ae. aegypti* range retraction in Australia has not been resolved, yet is probably related to a number of social improvement factors. In particular, a reduced prevalence of larval habitats (i.e. water-filled containers) due to improved reticulated water supplies and a concomitant decrease in domestic rainwater tanks, the decline of steam rail with its attendant water storage infrastructure and potential for dispersal, and, in rural areas in particular, the gradual replacement of domestic food storage cabinets (e.g. Coolgardie safes with their associated water containers) by kerosene and then electric refrigerators. Additionally, adult mosquito productivity and survival may have been reduced by greater yard sanitation with the advent of motor mowers limiting trash containers and adult resting sites and the development of residual insecticides (such as DDT, BHC and dieldrin) for domestic use. Furthermore, there was enhanced organization of vector control operations by local governments with the return in the late-1940s of well trained public and environmental health officers from military service who were rigorous in their destruction of breeding sites [10], and the relatively small human population sizes of *Ae. aegypti* infested areas in many parts of Australia may have facilitated extinction in some places. Finally, there may have been various biological factors that contributed, in some regions at least, to displacement or extinction, such as larval habitat competition from the indigenous ‘container mosquito’ *Aedes* (*Finlaya*) *notoscriptus* that was becoming domesticated and gradually spreading westwards in NSW from its native coastal habitats [10], [13].

However, many of these possible causes of *Ae. aegypti* range retraction remain speculative and not readily testable. We used computer-based simulation modelling to investigate why *Ae. aegypti* may have disappeared from much of its former range in Australia that appears still to be climatically favourable [14], [15] and to determine how well it may persist if reintroduced in the future.

Extinction processes have been previously studied through the use of mathematical models [16], [17]. In a recent application of computer-based modelling [18], a mechanistic approach was adopted to explain *Ae. aegypti* distribution in Australia, and described the historic range of the species in terms of its ability to survive in large breeding sites (rainwater tanks). This work demonstrated that large parts of coastal Australia could support survival of the species if such tanks were present, consistent with the historic range, but did not go as far as explaining range retraction. Furthermore, the model used by those authors made use of historic mean climate data, an approach that does not incorporate the stochasticity of daily weather variation that may contribute to extinction processes.

Here we describe the use of the Container Inhabiting Mosquito Simulation (CIMSiM) to determine the persistence of *Ae. aegypti* throughout Australia in its current and historic ranges. CIMSiM is a weather-driven depiction of the *Ae. aegypti* larval and adult habitat that describes the interaction between the mosquitoes and their environment [19], and has been validated for use in Australia [20]. In addition, we compared the performance of *Ae. aegypti* in terms of productivity throughout its range, examined the relative prevalence of life stages (i.e. eggs, larvae, adults) over time, and examined the relative prevalence of eggs in different habitats for selected localities. In doing this we hoped to explain its current range compared with its more extensive historic one in terms of climate suitability, and to comment on future risk of establishment in areas of Australia that are currently dengue free.

## Methods

### Lifetable simulation modelling with CIMSiM

CIMSiM [19], which accurately models *Ae. aegypti* population dynamics in Qld [20], generates daily estimates of egg, larval, pupal and adult numbers per hectare by integrating daily meteorological observations with information about available breeding habitats. Thirteen study locations were selected from both the current and historic Ae. aegypti range [10], [21]. Simulations were performed for 10 years (1998–2007). Model parameters for larval habitats [20] are provided (Table S1). All other model settings for CIMSiM were default values [19] with the exception of egg survivorship parameters which were modified (Table S1). The following daily weather observations were used: maximum, minimum and average daily temperature, relative humidity, saturation deficit, and rainfall. These were obtained for each study location from the Australian Bureau of Meteorology (www.bom.gov.au).

Ten simulations of 10 years length were performed for each location (with the exception of Harvey (WA), for which only six years of meteorological data were available), realising a total of 1170 simulated years. For each study location, we aimed to characterise the following performance measures for *Ae. aegypti*:

#### Productivity

Mean productivities for egg, larval and adult life stages were calculated for each locality, providing a measure of *Ae. aegypti* performance independent of extinction. This was done by 10 replicate 10 year simulations with random food delivery. Relative distribution of larvae and eggs between container types was simulated by a run with a fixed delivery of food producing 1 replicate of 10 years for each location.

#### Life-stage analysis

Each locality was assessed for whether a particular life-stage (egg, larva, adult) became absent at some point during the year. An average simulation was created for each locality, in which the average densities of each life-stage per hectare were calculated for each day of the 10 year simulation. This was done by 10 replicate 10 year simulations with random food delivery.

#### Persistence

Extinction was defined as the point at which all life stages (i.e. eggs, larvae, adults) were present at a density of <0.5/ha. Pupae were not considered due to the short duration of this stage. We chose 0.5/ha as the density required for extinction based on our understanding of typical densities [22] and dispersal [23] of *Ae. aegypti* in Australia. Egg survival rate parameters were adjusted for simulations to examine persistence (Text S1). This was done by 10 replicate 10 year simulations with random food delivery.

This examination of persistence involved application of constant container density and egg survival values in our simulations throughout Australia, which enabled us to determine the role of local climate. However, given that these parameters would not be constant in reality, we conducted a sensitivity analysis to determine the influence of changing container density and egg survival parameters on persistence. This was performed for two locations: Brisbane (historic range) and Charters Towers (current range). Persistence was evaluated by running single simulations with fixed food delivery at container densities (Table S1) increased and decreased by 20% respectively, and daily egg survival parameters altered +/−5%. This analysis was designed to inform us about how sensitive persistence was in relation to variation in these parameters. Results of this analysis are given in Table S4.

#### Immature habitat analysis

The persistence of eggs in each container type was determined through time to identify whether particular container types were critical to *Ae. aegypti* survival at a location. This was performed using a single simulation performed for each of three localities with fixed daily food delivery. This analysis was performed for a site in the current range, namely Cairns (Qld), and at two sites in the historic range: tropical Darwin (NT), and subtropical Brisbane (Qld).

## Results

### 

#### Productivity

Mean densities of *Ae. aegypti* eggs (Fig. 1), larvae and adults per ha. (Table S2) varied between locations. In general, higher densities were apparent in tropical regions (Fig. 1). Some locations outside the current range (Darwin, NT and Gosford, NSW) had higher mean densities than locations within it (notably Charters Towers, Qld).

#### Life-stage analysis

Within the current range (i.e. north Qld), all three life stages (egg, larvae, adults) of *Ae. aegypti* are present year round. However, in many parts of the southern historic range (south of and including Brisbane), populations are reduced to just eggs for part of the year. The exact duration of the egg-only period varied slightly with each year (Fig. S1), so the ranges for these periods have been given here (Fig. 1). The duration of the egg-only period lengthened with increasing latitude, with the southernmost location (Horsham, Victoria (Vic)) having egg-only periods for up to eight months of the year (Fig. 1).

#### Persistence

When simulations were performed with the egg survivorship settings in CIMSiM described above, extinction readily occurred at locations outside the current range (Fig. 1). For such locations this occurred in every replicate simulation, at a similar time-point (Table S3). Tennant Creek, NT (the site of an *Ae. aegypti* introduced infestation in 2004 that was ultimately eliminated by 2006) witnessed a single extinction recorded in 10 simulations. Within the historic range, extinction was not recorded at Darwin NT.

The sensitivity analysis revealed that persistence was sensitive to egg survivorship, with a 5% reduction in daily egg survival parameters leading to extinction at Charters Towers (current range). Conversely, a 5% increase led to survivorship in Brisbane, where extinction was previously predicted (historic range). Changes of +/−20% container densities had no observable influence on persistence (Table S4).

#### Immature habitat analysis

**Figure 2:**
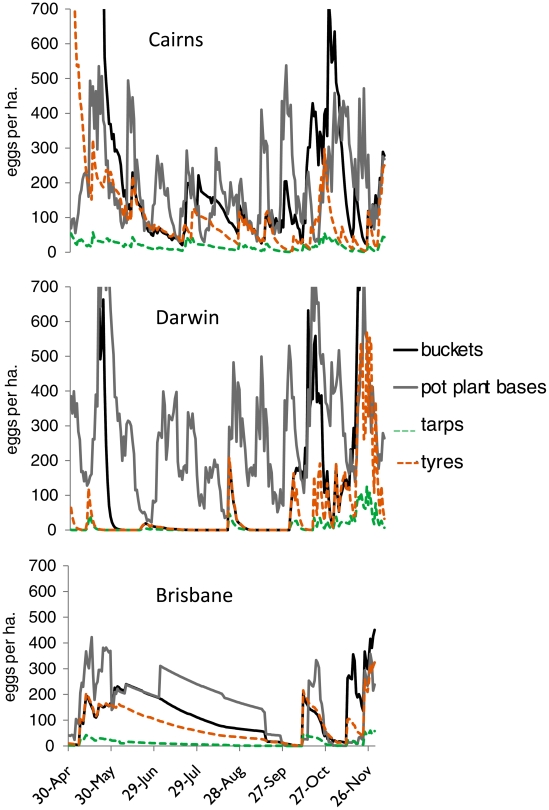
*Aedes aegypti* eggs per ha. in four simulated container types for May – Nov in Year 10 (2007) for one location in the current range (Cairns Qld) and two in the historic range (Darwin NT, Brisbane Qld).

For Cairns, Darwin and Brisbane, the density of *Ae. aegypti* eggs per ha. is reported for the four modelled container types during the period May – November 2007 inclusive (Simulation Year 10; Fig. 2), a period including the late austral autumn, winter and spring (tropical dry season). This time period was chosen as it coincides with the egg-only period for several localities (Fig. 1), and thus represents a potentially vulnerable period for the species.

At Cairns (within the current range), all four container types are active in terms of oviposition (Fig. 2), reflecting the year-round adult *Ae. aegypti* activity possible at these locations. However, at Darwin (within the historic range), a location at which *Ae. aegypti* is theoretically capable of strong productivity (Fig. 1), this activity is confined largely to pot plant saucers (continuously wet containers). In Brisbane, there is little new oviposition from July – mid-October, owing to conditions being too cool for adult activity (effective *Ae. aegypti* flight and mating does not typically occur below 15°C) [24].

## Discussion

### Explaining the current range of dengue vectors in Australia

The simulations described here have allowed a quantitative assessment of *Ae. aegypti* performance and persistence at localities inside and outside the current range in Australia. Coupled with information about the ecology of larval habitats derived here, explanations of the current and historic range of this disease vector in Australia are possible.

The continued presence of *Ae. aegypti* in its current range in Qld can be explained by its continuing year round adult and larval activity. This is facilitated by the continuous presence of suitable larval habitats, which remain wet enough (often due to constantly wet containers such as pot plant saucers in these simulations) and warm enough for year-round activity of all life stages (mean daily temperatures exceed 15°C year-round in the current north Qld range, www.bom.gov.au). An analysis of egg densities at Cairns throughout the dry season (May – Nov) (Fig. 2) revealed continued oviposition activity during this period.

Conversely, there are a number of factors which make the species vulnerable at localities in the historic range. In tropical Darwin where *Ae. aegypti* is now extinct, our modelling showed the species to be heavily reliant on manually-filled (i.e. continuously wet) containers for activity through the dry season. In our simulations, pot plant saucers (manually filled) were the major container type for eggs during the dry season. This represents vulnerability for *Ae. aegypti* in such locations, in that source reduction activities incorporating vector control and public education programs selectively targeting artificially flooded breeding sites could have a large negative impact on population growth. Kay and others [25] demonstrated that selective control of *Ae. aegypti* in continuously flooded subterranean wells in Qld reduced recolonization of surface containers during the wet season, reducing overall populations.

Such source reduction was evident in Darwin after World War II, when health officers returning from military service set about removal of rainwater tanks concomitant with the establishment of reticulated water supplies (Peter Whelan, NT Health Department, pers. comm. 22 Apr 2009). Rainwater tanks, while not manually filled, are preferentially filled from rainfall run-off and retain water for extended periods when naturally filled containers may have dried out. We were not able to simulate rainwater tanks here, which limits our ability to interpret their contribution to persistence. However, our inclusion of pot plant saucers (albeit much smaller but very productive sites) allow us to assess the importance of continuously wet larval habitats. Field productivity values for *Ae. aegypti* in rainwater tanks would be useful for future simulation modelling.

The importance of continuously wet containers for the persistence of *Ae. aegypti* in Darwin described here illustrates how vulnerable the species may have been when rainwater tanks were removed *en masse* post-war. Coupled with the small human population size in Darwin at the time (approx. 8016 people in the NT in 1948) [26], extinction by a combined action of habitat specificity and a lack of that habitat during the dry season due to source reduction is plausible. Insecticide application does not appear to have played a significant role in this process [11].

Thus, the local extinction of *Ae. aegypti* at Darwin was likely due to a synergistic combination of processes. This would have included a primary extinction driver (loss of habitat), combined with secondary drivers such as the specialisation of the species for a narrow range of habitat types and physiological vulnerability to dry conditions. Synergistic effects of extinction processes have been well studied for extinction dynamics in other species [27], [28]. Habitat and host specificity were both factors identified as being significant in the extinction of butterflies [16]. Such specificity is evident in *Ae. aegypti*, in its strong preference for artificial containers and blood-feeding on humans. While such associations promote the proliferation of the species in human habitats, they also render it vulnerable to changes in such habitat.

In modelling for Brisbane, the cool conditions through the austral winter were shown to preclude adult activity, making the species vulnerable in all habitats. Mean daily temperatures at this location are below the threshold for adult activity (15°C) [24] for June – Aug (www.bom.gov.au). Persistence is greatest in continuously wet containers. Considering the timing of its apparent extinction in Brisbane, during the 1950s, the decreasing prevalence of rainwater tanks concomitant with increased reticulated water supplies might explain the disappearance of *Ae. aegypti* from an area in which it was vulnerable to extinction.

Clearly, more than just strong seasonal productivity of *Ae. aegypti* is required for persistence at a location. This is evident in the very similar productivity values for the species in Brisbane (where extinction occurs in the model) and at Charters Towers (where it does not) (Table S2). In our modelling, the main difference between the locations is temperature, which is slightly higher at Charters Towers, permitting longer periods of adult activity and oviposition. This reduces extinction risk due to egg die-off as the egg-only periods are shorter (Fig. S1).

### Validating the model in terms of the historic range of dengue vectors

Here we confirm the ability of this species to survive in areas where it no longer exists (Fig. 1); a finding consistent with previous distribution reports [10]. However, by demonstrating extinction at some locations, our work challenges the idea that the historic range is climatically suitable for long-term *Ae. aegypti* survival as has been indicated [14], [15]. The former presence of *Ae. aegypti* in many parts of Australia is not questioned here; rather, the ability of CIMSiM to simulate strong periodic productivity in areas where the species was once considered seasonally common but is now extinct provides validation for our approach. The finding that *Ae. aegypti* passes several months of the year only as eggs (Fig. 1) is consistent with early field reports from southern temperate regions of NSW [29].

A number of possible factors for the retraction of the *Ae. aegypti* range in Australia have been suggested [10]. Each of these factors could have separately or in common with others plausibly reduced the size of local *Ae. aegypti* populations and the daily survivorship probability of adult mosquitoes, thereby contributing to local extinction. However, while the widespread introduction of town water reticulation in rural and regional areas has often been proposed as a crucial factor in the disappearance of *Ae. aegypti* from many southern localities/regions, many houses in many towns in these southern rural areas retained their water tanks throughout and following the period during which the mosquito disappeared, indicating there is no simple explanation that covers all situations.

The simulations performed here reveal that when adult daily survivorship probabilities are held high (0.91 in these simulations) and suitable breeding containers are available, *Ae. aegypti* is still vulnerable to extinction, particularly in southern Australia. In the first half of the 20^th^ Century, *Ae. aegypti* populations were widely sustained in southern Australia, no doubt with the aid of increased numbers of larval habitats and high adult survivorship probability. When these two factors became less prominent, the natural vulnerability of the species (as demonstrated by simulation here) could plausibly have led to extinction. Our sensitivity analysis revealed the importance of egg survivorship. Changes in the construction/materials of breeding containers over time (e.g. a greater proportion of plastic containers with time) could also have reduced egg survivorship rates.

### Limitations of our approach

We acknowledge that in applying a CIMSiM model field validated for north Qld across an entire continent we assume that *Ae. aegypti* performance in relation to temperature, humidity and rainfall remains constant. Furthermore, we also assume a constant breeding site diversity and density throughout Australia, and identical amounts of organic material (i.e. larval food) falling into containers. Naturally, we do not anticipate that in the field such generalities will hold true; there will almost certainly be some site-specific variation in local container-breeding mosquito ecology. However, such local scale differences would be very difficult to define accurately, and our approach in applying a constant set of model parameters at different locations (only differing with local meteorological data) was the only plausible way for us to model such a range of localities.

From our sensitivity analyses, we now understand that changing container densities by up to 20% is unlikely to influence persistence (at least using the containers simulated here). However, persistence is sensitive to changes in egg survivorship rates. Therefore, understanding how such rates are influenced in the field is critical for determining how persistence at a location may vary.

Furthermore, the relationship between ambient weather conditions and water temperature in the various container situations that form *Ae. aegypti* larval habitat has only recently become the focus of study in Australia [18]. The conversion factors for ambient to water temperature built into the CIMSiM program [19] accord well with actual observed temperatures in field-deployed tyres and buckets, albeit with some overestimation of maximum temperature on some days (MRK unpubl. data). Thus, when the water temperature is less than the thermal optimum for *Ae. aegypti* development, *Ae. aegypti* productivity in CIMSiM will be overestimated, and when above this threshold, productivity could be underestimated.

In addition, our choice of criterion for determining extinction at a location; densities of eggs, larvae and adults <0.5 per ha., could be scrutinized for the absence of pupae. Pupal densities were not included in the criterion, given the relatively short duration of this life stage (typically 1–3 d). However, it is possible (albeit improbable) that the pupal stage alone could facilitate persistence at a location when other life stages are at their nadir.

In applying the CIMSiM model so widely, we have assumed that on balance, our predictions of *Ae. aegypti* performance and persistence are satisfactory mid-range estimates that are useful for the kind of population-level analysis presented here.

### Comparison with other studies

Previous examination of *Ae. aegypti* range by climate-driven modelling indicated that this species could persist at locations in the historic range (such as Brisbane Qld and Darwin NT) in rainwater tanks (which always retained at least 1 cm of water depth), but not in small buckets, which frequently became dry [18]. Modelling of *Ae. aegypti* distribution using a genetic algorithm [21] also showed suitability of the historic range in the current climate. Our findings, in which *Ae. aegypti* eggs were most common in Darwin in manually-filled pot plant saucers during the dry season, were consistent with those of previous studies [18] which found that continuously wet habitats were required for persistence at this location.

### Future risk of dengue vector spread in Australia

Domestic water storage in tanks is increasing in southern Australia [30], and in the simulations presented here for southern locations (Harvey WA, Horsham Vic, Gosford and Wagga Wagga NSW, and Brisbane Qld), *Ae. aegypti* was reduced to existing as eggs only in continuously wet containers. Thus, any increase in water storage behaviour could improve the probabilities of survival of dengue vectors outside of its current range [21]. For this reason, the regulation of water storage behaviour to minimise mosquito breeding is crucial.

Areas of northern Australia where *Ae. aegypti* has become extinct (e.g. Darwin NT) remain vulnerable to re-establishment of the species, as evidenced by recent infestations at Tennant Creek and Groote Eylandt (NT). The absence of *Ae. aegypti* from these areas can only be maintained by adequate surveillance and source reduction activities targeted at manually filled containers (such as pot plant saucers) and domestic water storage.

According to our modelling, the introduction of a single cohort of *Ae. aegypti* into southern parts of the historic range in Australia is unlikely to result in a persistent population based on current climate, with container densities similar to that in the current range. Conversely, introductions into northern regions of the historic range (e.g. Darwin) may readily lead to persistence of the species.

### Controlling dengue globally

The failure of classical mosquito control methodologies (e.g. source reduction, insecticide application) for restricting dengue has stimulated the development of novel molecular strategies [2], [3]. While there is no doubt such strategies will be integral to the future of dengue control, the natural vulnerability of dengue vectors to extinction should not be forsaken. Incorporating extinction processes into integrated dengue control strategies in the future will ensure a greater probability of success. Furthermore, in subtropical and temperate regions where dengue is a problem, there may be no need for novel, biologically-engineered solutions.

## Supporting Information

Text S1 Simulation parameters.
(0.04 MB DOC)

Figure S1 Egg-only periods per year for localities where *Aedes aegypti* populations are reduced to just eggs for part of the year. Note: Simulation for Harvey (WA) ends Dec 31, 2003.
(5.12 MB TIF)

Table S1 CIMSiM model parameters which differ from default values provided by model developers (Focks et al.[8]).
(0.04 MB DOC)

Table S2 Mean productivity of *Ae. aegypti* life stages at simulated locations (SD in parentheses). Locations in the current (1990-) dengue transmission range are marked with an asterisk.
(0.04 MB DOC)

Table S3 Timing of extinctions of *Aedes aegypti* in 10 replicate simulations of 10 years (1998-2007). For most locations extinctions occurred in Year 1, with the exception of Tennant Creek (Year 9).
(0.04 MB DOC)

Table S4 Sensitivity analysis of container density and egg survivorship input parameters influence on persistence of *Aedes aegypti* at two locations, Brisbane and Charters Towers (1998-2007).
(0.03 MB DOC)

Alternative Language Abstract S1 Translation of the Abstract into Malay by Aishah Azil.
(0.03 MB DOC)

Alternative Language Abstract S2 Translation of the Abstract into Portugese by Mafalda Dias.
(0.03 MB DOC)
