# Supplementary material for: The Extinction of Dengue through Natural Vulnerability of Its Vectors
Source: PLoS Negl Trop Dis. 2010 Dec 21;4(12):e922. doi: 10.1371/journal.pntd.0000922 (PMC3006136; doi:10.1371/journal.pntd.0000922)
Supplement: Text S1 — Simulation parameters. (0.04 MB DOC) [file pntd.0000922.s001.doc]

**Text S1**

*Simulation parameters*

Archived meteorological data were obtained from the Bureau of Meteorology ([www.bom.gov.au](http://www.bom.gov.au/)). Within the current distribution, Cairns, Charters Towers, Innisfail, Mareeba and Townsville (all in Qld) were selected, and in the historic range Brisbane (Qld), Darwin (NT), Derby and Harvey (WA), Gosford and Wagga Wagga (NSW), and Horsham (Vic, the nearest station to Natimuk, the southernmost (but unconfirmed) locality record of *Ae. aegypti* in eastern Australia) were selected. Tennant Creek (NT), which is not in the historic nor current range but sustained an infestation of *Ae. aegypti* through at least one dry season between 2003 and 2005 [1,2], was also included (Fig. 1).

CIMSiM was initialised for use using the same parameter settings for each location (Supplementary material), with the exception of location-specific daily meteorological data. A recent 10 yr period (1998-2007) was chosen because of the high quality of recent data available and the range of rainfall and temperature conditions apparent over a period of such length. Daily observations of rainfall, temperature, relative humidity and saturation deficit were used.

Four container types were used for the simulations: i) 5L capacity plastic bucket (hereafter ‘buckets’), ii) pot plant saucers, iii) tarpaulins/plastic sheeting (‘tarps’) and iv) automobile tyres (‘tyres’). These container types were chosen because their water flux (filling and emptying rates) and *Ae. aegypti* productivity have been field validated for conditions in north Qld [3], and they represent a range of container sizes and properties that encompasses the majority of types commonly found in the field in north Qld Australia (P. Johnson pers. comm., James Cook Univ., Cairns, Qld., 2008). Furthermore, one of the container types, i.e. pot plant saucers, typically is manually filled with water, a function that can be selected in CIMSiM. In this way, while other container types require rainfall to be filled, pot plant saucers will always contain water, simulating the provision of larval habitat by humans in the absence of rainfall. Domestic rainwater tanks are a deliberate human water storage that can provide habitat for *Ae. aegypti* [4,5] and, while concern about their role in *Ae. aegypti* habitat provision has been stated [6], they remain an uncalibrated container type of uncertain productivity for this species. Thus, rainwater tanks were not included as a container here. The pot plant saucers, albeit of a different scale, are known to be a highly productive container type in northern Qld [7] (P. Johnson pers. comm., James Cook Univ., Cairns, Qld, 2008) that are manually filled by humans and fill the role of a continuously wet container in the simulations here.

# Descriptive settings for mosquito breeding containers (Table S1) were based on previously published field validations for north Qld [3]. The density of containers used in all simulations was constant, and was based on actual field densities from north Qld (P Johnson, James Cook University, unpubl. data 2008).

*Notes on egg survival rate parameters used in simulations*

To examine persistence, the egg survival parameters originally provided in CIMSiM [8] for these locations were altered by reducing each of the egg survival rates by 0.05 and each location was run with fixed food delivery over 10 years. Specifically, daily egg survival in wet containers was set at 0.95; in dry containers egg survival was set at 0.90 (for containers with sun exposure > 0.85) and 0.95 (for sun exposure ≤ 0.85); nominal egg survival at 0.95. These amendments were made as we considered the values to be unrealistically high based on our field observations of the species in Australia (CRW, SAR unpubl. obs.). Further, these values do not take biotic factors such as predation into account. Given the importance of egg survival in determining persistence at a location [5], we decided to amend the values downwards. These simulations were performed for locations in the current range (Cairns Qld), and the historic range (Brisbane Qld, Darwin and Tennant Creek NT, Harvey WA, Gosford NSW, and Horsham Vic) (Fig. 1).

**References**

1. Whelan PI, Krause V, Lamche G, N Kurucz (2004) *Aedes aegypti* mosquitoes, vectors of dengue found in Tennant Creek–elimination campaign in progress. NT Dis. Control Bull. 11: 1–3.

2. Whelan P, Pettit B, Krause V (2005) Dengue mosquito eradication project Tennant Creek. End of January 2005 progress report. NT Dis Control Bull 12: 1–3.

3. Williams CR, Johnson PH, Long SA, Rapley LP, Ritchie SA (2008) Rapid estimation of *Aedes aegypti* population size using simulation modeling, with a novel approach to calibration and field validation. J Med Entomol 45: 1173-1179.

4. Lee DJ, Hicks MM, Griffiths M, Debenham ML, Bryan JH, et al. (1987) *The Culicidae of the Australasian Region Volume 4*. Entomology Monograph No. 2. Canberra: Australian Government Publishing Service.

5. Kearney M, Porter WP, Williams C, Ritchie S, Hoffmann AA (2009) Integrating biophysical models and evolutionary theory to predict climatic impacts on species’ ranges: the dengue mosquito *Aedes aegypti* in Australia. Funct Ecol. doi: 10.1111/j.1365-2435.2008.01538.x

6. Beebe NW, Cooper RD, Mottram P, Sweeney AW. (2009) Australia’s Dengue Risk Driven by Human Adaptation to Climate Change. PLoS Negl Trop Dis 3: e429. doi:10.1371/journal.pntd.0000429

7. Kay BH, Barker-Hudson P, Hapgood GD, McCurley JO, Lyons GC et al. (1987) *Aedes aegypti* and dengue in the Townsville area, 1982-1985. Gen Appl Entomol 19, 2-10.

8. Focks DA, Haile DG, Daniels E, Mount GA. (1993) Dynamic life table model for *Aedes aegypti* (Diptera: Culicidae): analysis of the literature and model development. J Med Entomol 30: 1003-1017.

**Figure legend**

Fig. S1. Egg-only periods per year for localities where *Aedes aegypti* populations are reduced to just eggs for part of the year. Note: Simulation for Harvey (WA) ends Dec 31, 2003.
