# Supplementary figures and images for: The Extinction of Dengue through Natural Vulnerability of Its Vectors
Source: PLoS Negl Trop Dis. 2010 Dec 21;4(12):e922. doi: 10.1371/journal.pntd.0000922 (PMC3006136; doi:10.1371/journal.pntd.0000922)

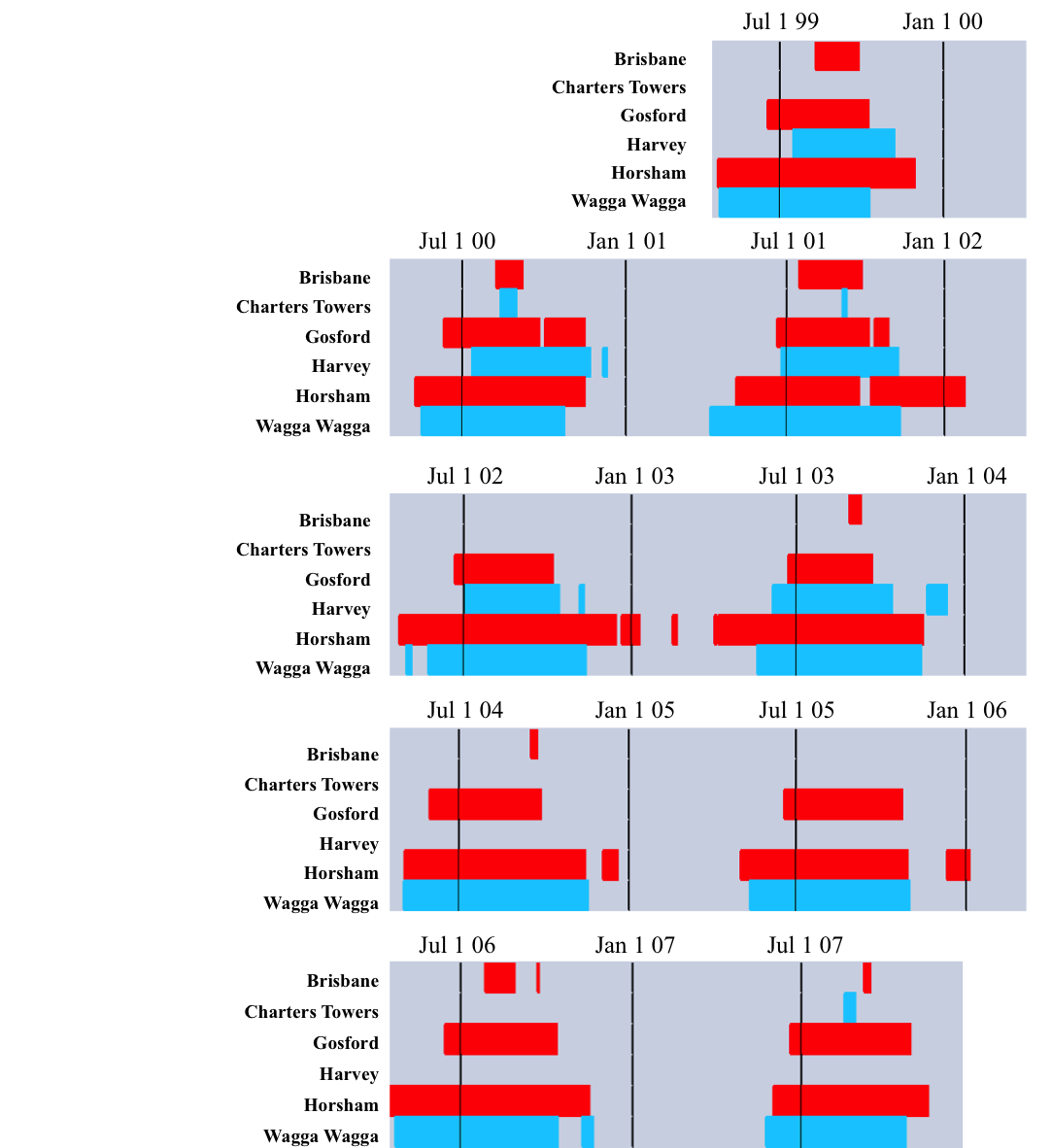

Supplement: Figure S1 — Egg-only periods per year for localities where Aedes aegypti populations are reduced to just eggs for part of the year. Note: Simulation for Harvey (WA) ends Dec 31, 2003. (5.12 MB TIF) [file pntd.0000922.s002.tif]
