# Supplementary material for: The Extinction of Dengue through Natural Vulnerability of Its Vectors
Source: PLoS Negl Trop Dis. 2010 Dec 21;4(12):e922. doi: 10.1371/journal.pntd.0000922 (PMC3006136; doi:10.1371/journal.pntd.0000922)
Supplement: Table S1 — CIMSiM model parameters which differ from default values provided by model developers (Focks et al.[8]). (0.04 MB DOC) [file pntd.0000922.s003.doc]

# Table S1. CIMSiM model parameters which differ from default values provided by model developers (Focks et al.^8^).

| Breeding container descriptive values (from Williams et al.^3^ ) | | | | |
| --- | --- | --- | --- | --- |
|  | Plastic buckets | Pot plant saucers | Tarpaulins | Tyres |
| Dimensions | 18cm d, 17.4 cm h | 6.1 x 2.0 x 26.0 cm | 15.5 x 22.6 x 4.2 cm | 35.3cm inner d, 11.0cm inner w |
| Capacity | 4871mL | 613mL | 520mL | 5343mL |
| Sun exposure | 0.2 | 0 | 0.8 | 0.3 |
| Container cover | 0 | 1 | 0 | 1 |
| Water shed ratio | 1.2 | 0 | 0.6 | 0.2 |
| Draw down (L) | 0 | 0.3 | 0 | 0 |
| Initial food (mg) | 70 | 100 | 450 | 500 |
| Mean food per d (mg) | 30 | 10 | 175 | 100 |
| density per ha. | 2.2 | 3.9 | 1.1 | 0.53 |
| Survivorship parameters | | | | |
| daily egg survivorship probability – wet containers | | | | 0.95 |
| daily egg survivorship probability – dry containers (sun > 0.85) | | | | 0.90 |
| daily egg survivorship probability – dry containers (sun < 0.85) | | | | 0.85 |
