# Supplementary material for: The Extinction of Dengue through Natural Vulnerability of Its Vectors
Source: PLoS Negl Trop Dis. 2010 Dec 21;4(12):e922. doi: 10.1371/journal.pntd.0000922 (PMC3006136; doi:10.1371/journal.pntd.0000922)
Supplement: Table S2 — Mean productivity of Ae. aegypti life stages at simulated locations (SD in parentheses). Locations in the current (1990-) dengue transmission range are marked with an asterisk. (0.04 MB DOC) [file pntd.0000922.s004.doc]

Table S2. Mean productivity of *Ae. aegypti* life stages at simulated locations (SD in parentheses). Locations in the current (1990-) dengue transmission range are marked with an asterisk.

| Location | mean eggs | mean larvae | mean adult females |
| --- | --- | --- | --- |
| Brisbane | 4172.3 (1240.2) | 2384.3 (738.5) | 99.7 (50.4) |
| Cairns* | 7725.2 (2078.1) | 8023.6 (1829.2) | 373.5 (110.4) |
| Charters Towers* | 3053.6 (2784.8) | 2607.4 (2416.9) | 104.8 (115.816) |
| Darwin | 12315.1 (15621.3) | 12496.5 (16728.2) | 686.5 (1018.4) |
| Derby | 4044.3 (5729.3) | 1253.3 (1205.4) | 78.9 (100.0) |
| Gosford | 6587.1 (6817.5) | 1234.5 (2047.4) | 57.1 (96.1) |
| Harvey | 702.5 (666.4) | 492.1 (751.2) | 14.4 (22.1) |
| Horsham | 253.1 (382.6) | 61.1 (204.1) | 2.6 (7.1) |
| Innisfail* | 64984.4 (79142.9) | 90173.4 (102552.3) | 3948.2 (5524.0) |
| Mareeba* | 4259.5 (4371.4) | 3736.6 (3947.2) | 149.5 (187.4) |
| Tennant Creek | 1890.3 (1914.3) | 1743.3 (1278.0) | 77.3 (66.8) |
| Townsville* | 4031.2 (4089.2) | 3542.1 (2936.8) | 163.5 (169.4) |
| Wagga Wagga | 708.9 (779.6) | 171.8 (341.5) | 6.6 (15.2) |
