# Supplementary material for: The Extinction of Dengue through Natural Vulnerability of Its Vectors
Source: PLoS Negl Trop Dis. 2010 Dec 21;4(12):e922. doi: 10.1371/journal.pntd.0000922 (PMC3006136; doi:10.1371/journal.pntd.0000922)
Supplement: Table S3 — Timing of extinctions of Aedes aegypti in 10 replicate simulations of 10 years (1998-2007). For most locations extinctions occurred in Year 1, with the exception of Tennant Creek (Year 9). (0.04 MB DOC) [file pntd.0000922.s005.doc]

Table S3. Timing of extinctions of *Aedes aegypti* in 10 replicate simulations of 10 years (1998-2007). For most locations extinctions occurred in Year 1, with the exception of Tennant Creek (Year 9).

| Location | Extinctions per 10 simulations | Timing of extinctions |
| --- | --- | --- |
| Brisbane | 10/10 | 21/9, 13/9, 23/9, 21/9, 14/9, 14/9, 15/9, 14/9, 13/9, 17/9 |
| Cairns | 0/10 | n.a. |
| Charters Towers | 0/10 | n.a. |
| Darwin | 0/10 | n.a. |
| Derby | 0/10 | n.a. |
| Gosford | 10/10 | 18/7, 20/7, 17/7, 20/7, 18/7, 21/7, 19/7, 18/7, 19/7, 17/7 |
| Harvey | 10/10 | 28/7, 6/8, 1/8, 26/7, 1/8, 23/7, 2/8, 7/8, 28/7, 31/7 |
| Horsham | 10/10 | 10/5, 10/5, 11/5, 10/5, 10/5, 10/5, 11/5, 10/5, 10/5, 10/5 |
| Innisfail | 0/10 | n.a. |
| Mareeba | 0/10 | n.a. |
| Tennant Creek | 1/10 | 3/9 |
| Townsville | 0/10 | n.a. |
| Wagga Wagga | 10/10 | 22/6, 20/6, 20/6, 19/6, 22/6, 24/6, 24/6, 18/6, 20/6, 20/6 |

# 
