# Supplementary material for: The Extinction of Dengue through Natural Vulnerability of Its Vectors
Source: PLoS Negl Trop Dis. 2010 Dec 21;4(12):e922. doi: 10.1371/journal.pntd.0000922 (PMC3006136; doi:10.1371/journal.pntd.0000922)
Supplement: Table S4 — Sensitivity analysis of container density and egg survivorship input parameters influence on persistence of Aedes aegypti at two locations, Brisbane and Charters Towers (1998-2007). (0.03 MB DOC) [file pntd.0000922.s006.doc]

# Table S4. Sensitivity analysis of container density and egg survivorship input parameters influence on persistence of Aedes aegypti at two locations, Brisbane and Charters Towers (1998-2007).

|  | container density | | egg survival | |
| --- | --- | --- | --- | --- |
|  | -20% | + 20% | -5% | + 5% |
| Brisbane | extinct – Year 1: 11/10 | extinct – Year 1: 12/10 | extinct - Year 1: 21/9 | no extinction |
| Charters Towers | no extinction | no extinction | extinct – Year 3: 22/9 | no extinction |
