# Supplementary material for: The Extinction of Dengue through Natural Vulnerability of Its Vectors
Source: PLoS Negl Trop Dis. 2010 Dec 21;4(12):e922. doi: 10.1371/journal.pntd.0000922 (PMC3006136; doi:10.1371/journal.pntd.0000922)
Supplement: Alternative Language Abstract S1 — Translation of the Abstract into Malay by Aishah Azil. (0.03 MB DOC) [file pntd.0000922.s007.doc]

**The extinction of dengue through natural vulnerability of its vector**

Ketiadaan kes demam denggi disebabkan oleh kelemahan semulajadi vektornya

Latarbelakang: Demam denggi ialah penyakit virus bawaan nyamuk yang paling utama di dunia. Untuk memastikan penyakit ini dikawal dengan berkesan pada masa hadapan, dinamik populasi bagi vektor terutamanya dalam konteks perubahan iklim, perlu difahami. Kemampuan kita untuk membuat ramalan tentang dinamik tersebut dicerminkan oleh kebolehan kita untuk menjelaskan taburan dan bilangan kes demam denggi dan vektornya menurut perubahan masa.

Kaedah kajian/Hasil kajian: Kami menggabungkan rekod cuaca harian dan teknik pemodelan simulasi untuk menerangkan kewujudan vektor [*Aedes aegypti* (L.)] mengikut taburannya yang semasa dan terdahulu. Kami membuktikan bahawa di kawasan di mana demam denggi dilaporkan pada masa kini di Australia (kawasan Wet Tropics, Far North Queensland), keadaan adalah sesuai bagi aktiviti Ae. aegypti dewasa dan oviposisi untuk sepanjang tahun. Walau bagaimana pun, pada masa lalu, vektor terdedah kepada kepupusan berkala disebabkan oleh sekatan kepada aktiviti nyamuk dewasa dan kehilangan tempat pembiakan yang sesuai secara rawak.

Kesimpulan/Kepentingan kajian: Hasil kajian ini apabila digabungkan dengan perubahan tingkahlaku manusia dalam menyimpan bekalan air, boleh menjelaskan taburan vektor yang semakin berkurangan dengan perjalanan masa.Oleh yang demikian, penghapusan demam denggi di kawasan tropika pada masa hadapan akan menjadi sukar jika hanya kawalan vektor konvensional digunakan . Namun demikian, kawalan *Ae. aegypti* di kawasan beriklim sederhana dan subtropika akan sangat dipengaruhi oleh polisi kerajaan yang berkaitan dengan bekalan air domestik. Pengetahuan tentang kelemahan semulajadi vektor (e.g. habitat yang khusus, pengaruh iklim) seharusnya dimanfaatkan dan digabungkan ke dalam teknik transgenik dan bakteria simbiotik yang baru berkembang; untuk membangunkan strategi kawalan dan penghapusan demam denggi untuk masa hadapan.
