# Supplementary material for: The Extinction of Dengue through Natural Vulnerability of Its Vectors
Source: PLoS Negl Trop Dis. 2010 Dec 21;4(12):e922. doi: 10.1371/journal.pntd.0000922 (PMC3006136; doi:10.1371/journal.pntd.0000922)
Supplement: Alternative Language Abstract S2 — Translation of the Abstract into Portugese by Mafalda Dias. (0.03 MB DOC) [file pntd.0000922.s008.doc]

Resumo

Introdução: Das doenças causadas por vírus e transmitidas através de mosquitos, dengue é a mais important no mundo. O sucesso no futuro, para o control desta doença, requer uma compreenção das dinâmicas populacionais do inseto transmissor, principalmente no que diz respeito às mudanças climáticas. A nossa capacidade de prever estas dinâmicas no futuro, é reflectida na nossa habilidade em explicar as importantes mudanças históricas na distribuição e abundância desta doença e do seu inseto transmissor.

Metodologia/Principais achados: Aqui, nós combinamos o registo diário do clima com técnicas de modelagem de simulação para explicar a persistência do inseto transmissor (*Aedes aegypti* (L.)) dentro da sua actual e histórica area na Australia. Nos verificamos que, nas regiões onde dengue ocorre presentemente na Australia (na regiõe trópical húmida do extremo Norte do Queensland), as condições são persistentemente apropriadas para a actividade e oviposição do adulto Ae. aegypti ao longo de todo o ano. Contudo, nas áreas históricas, o inseto transmissor é vulneravel à extinção periódica devido à combinada influêcia de restrições da actividade adulta e a perca aleatória de sítios apropriados para oviposição.

Conclusão/Significado: Estes resultados, juntamente com mudanças na maneira de armazenar água pelas pessoas, pode explicar a observada diminuição da area histórica do inseto transmissor da doença. Por estas razões, a futura erradicação de dengue nas regiões trópicais húmidas será extremamente difícil somente com meios clássicos de controlar mosquitos. No entanto, o control de *Ae. aegypti* em regiões sub-trópicais e temperadas será imensamente facilitado através de programas governamentais que regulem o armazenamento da agua a nível doméstico. A exploração das vulnerabilidades naturais de insetos transmissors de dengue (ex: especificação do habitat, limitações climáticas) deveriam ser integradas com o aparecimento de novas técnicas transgênicas e técnicas pertinentes à simbiose para o control da bacteria, em ordem a desenvolver o futuro control e as futuras estratégicas de eliminação do transmissor.
